# Power Generation Performance of Reverse Electrodialysis (RED) Using Various Ion Exchange Membranes and Power Output Prediction for a Large RED Stack

**DOI:** 10.3390/membranes12111141

**Published:** 2022-11-13

**Authors:** Yu Sugimoto, Ryo Ujike, Minato Higa, Yuriko Kakihana, Mitsuru Higa

**Affiliations:** 1 Graduate School of Science and Technology for Innovation, Yamaguchi University, 2-16-1 Tokiwadai, Ube, Yamaguchi 755-8611, Japan; 2 Blue Energy Center for SGE Technology (BEST), Yamaguchi University, 2-16-1 Tokiwadai, Ube, Yamaguchi 755-8611, Japan

**Keywords:** salinity gradient energy, reverse electrodialysis, ion exchange membrane, power output prediction

## Abstract

Reverse electrodialysis (RED) power generation using seawater (SW) and river water is expected to be a promising environmentally friendly power generation system. Experiments with large RED stacks are needed for the practical application of RED power generation, but only a few experimental results exist because of the need for large facilities and a large area of ion-exchange membranes (IEMs). In this study, to predict the power output of a large RED stack, the power generation performances of a lab-scale RED stack (40 membrane pairs and 7040 cm^2^ total effective membrane area) with several IEMs were evaluated. The results were converted to the power output of a pilot-scale RED stack (299 membrane pairs and 179.4 m^2^ total effective membrane area) via the reference IEMs. The use of low-area-resistance IEMs resulted in lower internal resistance and higher power density. The power density was 2.3 times higher than that of the reference IEMs when natural SW was used. The net power output was expected to be approximately 230 W with a pilot-scale RED stack using low-area-resistance IEMs and natural SW. This value is one of the indicators of the output of a large RED stack and is a target to be exceeded with further improvements in the RED system.

## 1. Introduction

Recently, salinity gradient energy (SGE), which is generated when two salt solutions with different concentrations are mixed, has attracted attention as a renewable energy source. Reverse electrodialysis (RED) is a technology that converts SGE into electrical energy using ion exchange membranes (IEMs) [1,2]. RED power generation uses the electrochemical potential created by IEMs to generate electricity and has access to a nearly inexhaustible supply of seawater (SW) and river water (RW), thus eliminating issues regarding depletion of energy sources. As RED power generation is largely unaffected by weather conditions and has a high operating rate, it has great potential as a new power generation method.

A RED stack comprises alternating layers of cation exchange membranes (CEM) and anion exchange membranes (AEM) between two electrodes. Gaskets with spacer nets are placed between the membranes to maintain a constant distance between the membranes, create a flow path, and agitate the solutions. An electric potential difference is generated by alternately supplying high- and low-concentration solutions between the membranes. When an external load resistance is connected to the two electrodes, ionic diffusion from the high-concentration side to the low-concentration side is converted to an electric current by a redox reaction at the electrodes; hence, the RED stack generates electricity, i.e., converts SGE to electricity.

In REDs, where ion diffusion plays a leading role, the selective transport of ions through IEMs is important for power generation in the RED system [3]. Therefore, evaluations of small (lab-scale) RED stack power generation performance using various membranes have been conducted [4,5,6,7,8,9,10,11,12,13,14,15,16,17,18,19,20,21,22]. Even if limited to commercial membranes, Neosepta ^®^ CMX, AMX (Astom Corp., Yamaguchi, Japan) [6,12,14,18,21,22], Fuji V1, Fuji V2, Fuji-CEM, Fuji-AEM (FUJIFILM Manufacturing Europe B.V., Tilburg, The Netherlands) [10,11,15,18,19,20,21,22], Selemion^®^ CSO, AMV (AGC Inc., Tokyo, Japan) [6,18,21], Fumasep^®^ FKS, FAS, (FUMATECH BWT GmbH, Bietigheim-Bissingen, Germany) [5,6,10], PC-SK, PC-SA (PC Cell GmbH, Heusweiler, Germany) [17], Ralex^®^ CMH, AMH (MEGA AS, Straz Pod Ralskem, Czech Republic) [6,7], and many other commercial membranes have been tested. However, performance evaluations with large (pilot-scale) RED stacks are required for practical use. Tedesco et al. have demonstrated 65 W with 5 M NaCl and 0.03 M NaCl in the RED stack with 125 CEM PR1/AEM PR1 (FUJIFILM Manufacturing Europe B.V., The Netherlands, with a total effective membrane area of 48.4 m^2^) membrane pairs [23] and 309 W with 215 mS cm^−1^ NaCl and 3.5 mS cm^−1^ NaCl in the RED stack with 500 CEM PR1/AEM PR1 (with a total effective membrane area of 193.6 m^2^) membrane pairs [24]. Nam et al. achieved 309 W with natural SW and treated wastewater in the RED stack with 1000 CEM Type-I/AEM Type-I (FUJIFILM Manufacturing Europe B.V., The Netherlands, with a total effective membrane area of 250 m^2^) membrane pairs [25]. Mehdizadeh et al. achieved 39 W with 53 mS cm^−1^ NaCl and 1.3 mS cm^−1^ NaCl in the RED stack with 200 Neosepta^®^ CMX/AMX (Astom Corp., Japan, with a total effective membrane area of 40 m^2^) membrane pairs [26] and 263 W with 1 M NaCl and water treatment plant surface water (0.34 mS cm^−1^) in the RED stack with 299 Neosepta^®^ CIMS/ACS-8T (Astom Corp., Japan, with a total effective membrane area of 179.4 m^2^) membrane pairs [27]. Although it is important to compare the performance of RED stacks using different IEMs with different properties, it is difficult to evaluate the relationship between membrane properties such as membrane resistance, ion selectivity, and RED performance in pilot-scale RED systems with large membrane areas. Therefore, only a few studies have been conducted on the performance of pilot-scale RED stacks using various types of IEMs. We evaluated the performance of the pilot-scale RED stack using the CIMS and ACS-8T [27]. By evaluating the performance of lab-scale stacks using various commercial IEMs, including CIMS/ACS-8T, and determining the performance ratio between commercial CEMs/AEMs and CIMS/ACS-8T, we can estimate the pilot-scale RED performance using these IEMs.

The objective of this study is to predict the power output of a pilot-scale RED stack with various commercial IEMs from experiments using a lab-scale RED stack. We evaluated and analyzed the power generation performance of a lab-scale RED stack with 40 membrane pairs and a total membrane area of 7040 cm^2^ using five types of CEM/AEMs with different membrane properties, including reference membranes; CIMS and ACS-8T. Model SW, model concentrated SW (RO brine), and natural SW were used as feed solutions. To discuss the differences in the open-circuit voltage of the lab-scale RED stack, the membrane potential of each IEM was measured. This study suggests that the output of pilot-scale RED could be more than doubled by changing CIMS/ACS-8T to IEMs with low-area-resistance.

## 2. Experimental Section

### 2.1. Membranes

CEM and AEM pairs: Neosepta^®^ CSE/ASE, Neosepta^®^ CMX/AMX, Neosepta^®^ CIMS/ACS-8T, C-2/A-2 (ASTOM Corp., Japan), and Fumasep^®^ FKS-20/FAS-20 (FUMATECH BWT GmbH, Germany) were used as CEM/AEM for lab-scale RED stacks. CSE/ASE and CMX/AMX are the current- and previous-generation standard membranes, respectively. The CIMS/ACS-8T are monovalent ion-selective membranes. C-2/A-2 and FKS-20/FAS-20 exhibited low-area-resistance. Membrane properties were measured in the same way as previously described [28,29]. The basic properties of the membranes used in this study are listed in Table 1. For the monovalent ion-selective membranes, CIMS and ACS-8T, the membrane potentials were measured with the selective side facing the high-concentration solution. For all basic membrane properties and membrane potential measurements, all reagents were of analytical grade, and solutions were prepared using ion-exchanged water.

### 2.2. Solutions for RED Power Generation Test

A 50 mS cm^−1^ (≈0.516 M, M = mol dm^−3^) NaCl solution and 90 mS cm^−1^ (≈1.18 M) NaCl solution were used as model SW and model RO brine, respectively, because we had used RO brine from Mamizu Pia (Fukuoka, Japan) in a previous study, and its conductivity was approximately 90 mS cm^−1^ [30]. 0.30, 0.45, 1.50, 2.24, and 3.40 mS cm^−1^ (≈2.3, 3.7, 13.5, 20.3, and 31.1 mM) NaCl solutions were used as model river water (RW). To evaluate the power generation performance of the RED stack using actual solutions, SW collected at an SW desalination plant (Chatan, Okinawa, Japan) was used as the high-concentration solution (HS) and surface water from a water treatment plant (Chatan, Okinawa, Japan) was used as the low-concentration solution (LS). The electrical conductivity and ionic composition of natural SW and surface water are listed in Table 2. Natural SW and surface water were filtered using a filtration system (AF-4, ZEOLITE Co. Ltd., Fukuoka, Japan) to remove impurities before use. Surface water was adjusted to conductivities of 0.45, 1.50, 2.24, and 3.40 mS cm^−1^ by adding natural SW. 3.0 M NaCl solution was used as the electrode solution in both the model and natural solutions. All NaCl solutions used for RED power generation tests were prepared using 95% NaCl purchased from Diasalt Corporation (Nagasaki, Japan) and tap water.

### 2.3. Construction of the Lab-Scale RED Stack

**Figure 1:**
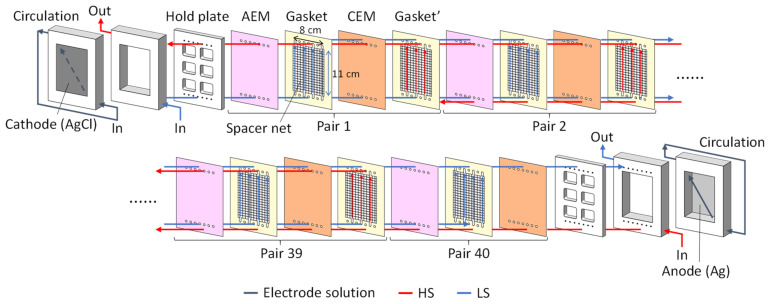
Schematic illustration of the lab-scale RED stack.

Figure 1 shows a schematic of the lab-scale RED stack. Forty pairs of CEMs/AEMs were used for the RED stack. The monovalent ion-selective membranes, CIMS, and ACS-8T were stacked alternatively with the ion-selective surface facing the HS. The effective area of the membrane in contact with the solution per IEM was 88 cm^2^ (11 cm × 8 cm). The total effective membrane area of the RED stack was 7040 cm^2^ (88 cm^2^ × 80). To maintain the inter-membrane distance and prevent solution leakage, a gasket with an integrated spacer of 200 μm thickness was placed between the CEM and AEM. Ag and AgCl were used as cathode and anode, respectively.

### 2.4. Evaluation of Power Generation Characteristics

Figure S1 shows a flow diagram of the RED stack performance evaluation. Solutions were fed to the RED stack using a magnet pump (MD-30RZ-N, IWAKI Co., Ltd., Tokyo, Japan), and the linear flow velocity (*v*_LS_) in the LS channel inside the RED was set to 2.0 cm s^−1^ (0.77 L min^−1^). The linear flow velocity was calculated using the following equation:

(1) $$v_{\mathrm{LS}}=\frac{Q_{\mathrm{LS}}}{dLN}$$

where *Q*_LS_ is the flow rate in the LS channel, *d* is the distance between membranes (200 μm), *L* is the width of the flow channel (8 cm), and *N* is the number of membrane pairs (40). The flow rate of the HS channel was set such that the pressure at the HS channel inlet was 3 kPa higher than that at the LS channel inlet. The pressures of the HS and LS channels were measured using a digital pressure gauge (KDM30, Krone Corporation, Tokyo, Japan).

An electronic load device (PLZ164W, Kikusui Electronics Corporation, Kanagawa, Japan) was connected to the electrodes of the RED stack, and the current (*I*) was varied from 0 A at a rate of 0.2 A min^−1^ to measure the voltage (*V*)–*I* curve. *V* in the RED stack is expressed as follows:

(2) $$V=V_{\mathrm{OC}}-R_{\mathrm{int}}I$$

where *V*_OC_ is the open-circuit voltage and *R*_int_ is the internal resistance of the RED stack. *V*_OC_ is defined as the voltage at zero current in the *V*-*I* curve. Contrastingly, assuming that the intramembrane diffusion potential is negligible, *V*_OC_ can be calculated from the Nernst equation [31].

(3) $$V_{\mathrm{OC},\;\mathrm{theory}}=\frac{2t_{\mathrm{ave}}NRT}{F}\ln\left(\frac{\gamma_{\mathrm{HS}}c_{\mathrm{HS}}}{\gamma_{\mathrm{LS}}c_{\mathrm{LS}}}\right)$$

(4) $$t_{\mathrm{ave}}=\frac{t_{\mathrm{CEM}}+t_{\mathrm{AEM}}}{2}$$

where *t*_CEM_ and *t*_AEM_ are the transport numbers of the CEM and AEM, respectively; *t*_ave_ is the average of the transport numbers of CEM and AEM; *R* is the gas constant (8.314 J mol^−1^ K^−1^); *T* is the absolute temperature; *F* is the Faraday constant (96,485 C mol^−1^); *γ*_HS_ is the activity coefficient of NaCl in HS; *γ*_LS_ is the activity coefficient of NaCl in LS; *c*_HS_ is the concentration of NaCl in HS; and *c*_LS_ is the concentration of NaCl in LS. *V*_OC, theory_ was calculated using *T* = 298 K and *t*_ave_ = 0.98, and the activity coefficient values from Table S1. Although *R*_int_ is ideally obtained from the slope of the *V*-*I* curve (see Equation (2)), the slope of the actual *V*-*I* curve in the RED performance test was not constant and decreased with increasing current because the higher the current, the higher the conductivity of the solution on the LS side owing to ionic diffusion. Therefore, *R*_int_ was defined in this study as the slope of the *V*-*I* curve at the current value when the maximum output (*P*_max_) was obtained. From Equation (2), and P=VI, *P*_max_ is given by [32]

(5) $$P_{\max}=\frac{V_{\mathrm{OC}}{}^{2}}{4R_{\mathrm{int}}}$$

The maximum output density (*PD*_max_), which is the maximum output per membrane area, was calculated using the following equation:

(6) $$PD_{\max}=\frac{P_{\max}}{2AN}$$

where A is the effective membrane area (88 cm^2^).

## 3. Results and Discussion

### 3.1. Model SW and Model RW

#### 3.1.1. Comparison of the Open Circuit Voltage

The open-circuit voltage (*V*_OC_) and internal resistance of the RED stack (*R*_int_) are important factors for analyzing the performance of the RED stack. The conductivity of SW or RO brine as an HS depends on the site location of the RED plant. Therefore, examining the effect of conductivity at the LS channel on *V*_OC_ and *R*_int_ is an important factor when considering the operating conditions for obtaining a high RED output.

**Figure 2:**
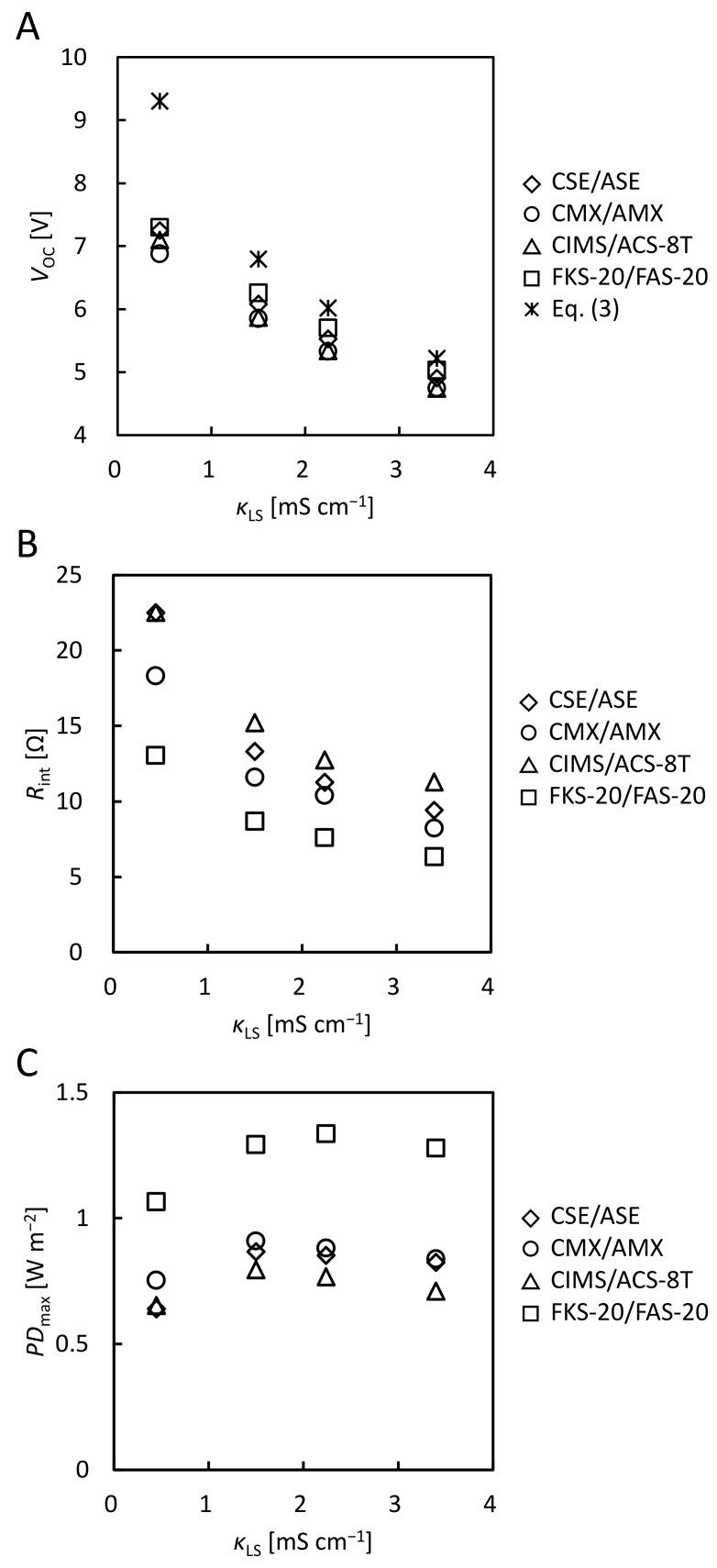
*κ*_LS_ dependence of (A) *V*_OC_, (B) *R*_int_, and (C) *PD*_max_ using model SW (50 mS cm^−1^ NaCl) as HS and model RW as LS.

Figure S2 shows the *V*-*I* curves of the RED stacks with various membrane pairs using model SW and model RW. Figure 2A shows the LS conductivity (*κ*_LS_) dependence of *V*_OC_ obtained from the voltage at zero current in these *V*-*I* curves. For all IEM pairs, *V*_OC_ decreased as *κ*_LS_ increased because the concentration ratio *c*_HS_/*c*_LS_ in Equation (3) decreased with an increase in *κ*_LS_. The *V*_OC_ increased in the following IEMs pair order: CMX/AMX < CIMS/ACS-8T < CSE/ASE < FKS-20/FAS-20. *V*_OC_ per pair of CIMS/ACS-8T was 0.18, 0.15, 0.13, and 0.12 V at *κ*_LS_ = 0.45, 1.50, 2.24, and 3.40 mS cm^−1^, respectively. In previous studies, *V*_OC_ per CIMS/ACS-8T pair using a pilot-scale RED stack at various feed flow rates was 0.14~0.17 V using 50 mS cm^−1^ NaCl as HS and 0.34 mS cm^−1^ surface water as LS [27]. The *V*_OC_ per CIMS/ACS-8T pair using the lab-scale RED stack in this study showed almost the same values as those obtained using the pilot-scale RED stack.

The experimental values of *V*_OC_ for all IEM pairs were lower than the values calculated from Equation (3) for all the *κ*_LS_ values. In particular, *V*_OC_ was much lower than *V*_OC,theory_, at a smaller *κ*_LS_. To examine the cause of the deviation between *V*_OC_ and *V*_OC,theory_, the membrane potential of a diffusion dialysis system consisting of an IEM, and 50 and 1.5 mS cm^−1^ NaCl solution was measured, as shown in Figure S3A. The absolute values of the membrane potential were approximately 88 mV for all the CEMs and 80 mV for all the AEMs. In previous studies, the absolute value of the membrane potential of CMX was higher than that of AMX [33,34]. The difference in the absolute values of the membrane potential between CEM and AEM is expected to be due to the junction potential between the salt bridges (3 M KCl) and the measurement solutions (50 and 1.5 mS cm^−1^ NaCl solutions) [29]. The membrane potential generated when 40 pairs of CEMs/AEMs are stacked (total *ϕ*_m_), can be calculated using the following equation:

(7) $$\mathrm{Total}\;\phi_{m}=40\left(\left|\phi_{m,\mathrm{CEM}}\right|+\left|\phi_{m,\mathrm{AEM}}\right|\right)$$

where *ϕ*_m,CEM_ and *ϕ*_m,AEM_ are the membrane potentials of the CEM and AEM, respectively. The calculated total *ϕ*_m_ is shown in Figure S3B. The calculated values of all IEM pairs used in this study were slightly lower than the ideal membrane potential (≈6.8 V) calculated using Equation (3). Here, the junction potential mentioned above was canceled out when |*ϕ*_m,CEM_| and |*ϕ*_m,AEM_| were added together. There were few differences in the total *ϕ*_m_ between the IEM pairs. This indicates that the effect of the difference in the membrane properties listed in Table 1 on the membrane potential is small under the measurement conditions. The deviation between the total *ϕ*_m_ (≈6.8 V) and *V*_OC_ of the stack using the IEMs pairs at *κ*_LS_ = 1.50 mS cm^−1^ (5.9~6.3 V) was not negligible. The possible causes of the deviation are as follows: In the case of the membrane potential in the diffusion dialysis system, there is no spacer on the membrane surfaces, and the solutions in contact with the membrane were well stirred. However, there is a spacer on the membrane surfaces in the feed channels of the RED stack. Hence, the effect of concentration polarization on the decrease in the membrane potential in the stack [27] will be higher than that in the diffusion dialysis system. FKS-20/FAS-20 had the highest *V*_OC_ among all CEM/AEM pairs, indicating that FKS-20/FAS-20 may have the lowest osmotic water flow, which is a cause of the decrease in the membrane potential due to the external concentration gradient.

#### 3.1.2. Comparison of the Internal Resistance

The *V*-*I* curves in Figure S2 show that the slope is not straight and decreases as the current increases, although the *V*-*I* curve is theoretically straight (see Equation (2)). This is due to the increase in the concentration of the LS channel in the stack because of the diffusion of ions from the HS to the LS when an electric current is generated, resulting in a decrease in the *R*_int_ of the RED stack. Hence, in this study, *R*_int_ was defined as the slope of the *V*-*I* curve at the current when *P*_max_ was obtained, as described in Section 2.4.

Figure 2B shows the *κ*_LS_ dependence of *R*_int_. For all the IEM pairs, *R*_int_ decreased as *κ*_LS_ increased, which was due to the decrease in LS resistance. The value of *R*_int_ ranged from 6 Ω to 23 Ω. In a previous study, the RED stack with a comparable effective membrane area (100 cm^2^ × 50 FKD/FAD (FUMATECH BWT GmbH, Bietigheim-Bissingen, Germany) pairs = 10,000 cm^2^ and 200 μm inter-membrane distance) using 30 g NaCl L^−1^ (≈0.51 M NaCl) as HS and 1 g NaCl L^−1^ (≈0.017 M NaCl) as LS showed a *R*_int_ value of approximately 18 Ω [35]. The stack had the same inter-membrane distance (200 μm) and almost the same membrane resistance (CEM; FKD 2.14 Ω cm^2^ and AEM; FAD 0.89 Ω cm^2^ [6]) as those of our stack. Hence, the stack resistance per pair of CEM/AEM (0.345 Ω) [6] was almost the same as that of our stack (0.15–0.575 Ω). The *R*_int_ increased in the following IEM pairs order: FKS-20/FAS-20 < CMX/AMX < CSE/ASE CIMS/ACS-8T. The stack using FKS-20/FAS-20 had the lowest *R*_int_ among all the stacks. This is attributed to the lowest area resistance of FKS-20/FAS-20 in all IEM pairs. In contrast, the highest *R*_int_ value was obtained when the CIMS/ACS-8T pair was used. In previous studies, the *R*_int_ of CIMS/ACS-8T was higher than that of CMX/AMX [29], which is consistent with the current tendency. This will be due to the fact that membrane resistance was measured by the alternating current (AC) method; on the other hand, direct current was generated in a RED stack. Owing to the monovalent ion-selective surface of CIMS and ACS-8T, the resistance was higher for direct current than for alternating current.

The RED stack comprised one electrode pair and many cell pairs. The cell comprised the flow channels of the HS, LS, CEM, and AEM. Hence, to analyze the dependence of the electrical resistance of each component on *R*_int,_ the internal resistance of the RED stack (*R*_int,cal,_) was calculated from the summation of the resistances due to the electrode, HS, LS, CEM, and AEM, including the spacer shadow effect [36]:

(8) $$R_{\mathrm{int},\;\mathrm{cal}}=R_{\mathrm{el}}+(R_{\mathrm{HS}}+R_{\mathrm{LS}}+R_{\mathrm{CEM}}+R_{\mathrm{AEM}})$$

(9) $$R_{\mathrm{HS}}=\frac{\beta_{\mathrm{sol}}Nd}{A\kappa_{\mathrm{HS}}}$$

(10) $$R_{\mathrm{LS}}=\frac{\beta_{\mathrm{sol}}Nd}{A\kappa_{\mathrm{LS}}}$$

(11) $$R_{\mathrm{CEM}}=\frac{\beta_{\mathrm{mem}}NR_{A,\;\mathrm{CEM}}}{A}$$

(12) $$R_{\mathrm{AEM}}=\frac{\beta_{\mathrm{mem}}NR_{A,\;\mathrm{AEM}}}{A}$$

where *R*_el_ is the electrode resistance; *R*_HS_ and *R*_LS_ are the resistances of the channels at HS and LS, respectively; *R*_CEM_ and *R*_AEM_ are the resistances of the CEM and AEM, respectively; *β*_sol_ is the spacer shadow effect in the solution compartment (1.93); *β*_mem_ is the spacer shadow effect on the membrane (1.10); *κ*_HS_ is the conductivity of HS. Details of the calculation of *β*_sol_ and *β*_mem_ are provided in the Supplementary Materials.

Figure S4 shows the *κ*_LS_ dependence of *R*_int,cal_. For all IEM pairs at all values of *κ*_LS_, *R*_LS_ occupied the highest proportion of *R*_int,cal_. The second highest proportion in *R*_int,cal_ was *R*_CEM_ + *R*_AEM_, and the contribution of *R*_el_ and *R*_HS_ to *R*_int,cal_ was low. The experimental values at *κ*_LS_ = 0.45 mS cm^−1^ were lower than *R*_int,cal_ values in all IEM pairs. As mentioned above, the conductivity of the LS channel inside the RED cells will be higher than that of the LS channel inlet because of ionic diffusion via the IEMs. Simultaneously, the conductivity of the HS channel decreases; however, the contribution of *R*_LS_ is much larger than that of *R*_HS_. Therefore, *R*_int_ was lower than *R*_int,cal_ due to the increase in the conductivity of the LS channel. At *κ*_LS_ ≥ 1.50 mS cm^−1^, the *R*_int_ values of CSE/ASE, CMX/AMX, and FKS-20/FAS-20 were lower than *R*_int,cal_, while those of CIMS/ACS-8T were almost the same as *R*_int,cal_. This will be due to the fact that the former three IEM pairs had lower resistance than CIMS/ACS-8T (see Figure 2B); hence, the RED stack had a higher electric current than CIMS/ACS-8T. Owing to the high current, the resistance of the LS channel is lower than that calculated.

#### 3.1.3. Comparison of the Maximum Power Density

Figure 2C shows the *κ*_LS_ dependence of the *PD*_max_. For CSE/ASE, CMX/AMX, and CIMS/ACS-8T, *PD*_max_ was maximal around *κ*_LS_ = 1.50 and 2.24 mS cm^−1^. For FKS-20/FAS-20, *PD*_max_ was maximal around *κ*_LS_ = 2.24, and 3.50 mS cm^−1^. This is due to the decrease in *V*_OC_ and *R*_int_ with increasing *κ*_LS_ (see Equation (5)). *PD*_max_ increased in the following IEM pairs order: CIMS/ACS-8T < CSE/ASE < CMX/AMX < FKS-20/FAS-20. The small *PD*_max_ in CIMS/ACS-8T is due to the small *V*_OC_ and high *R*_int_, while the large *PD*_max_ in FKS-20/FAS-20 is due to the large *V*_OC_ and low *R*_int_. 1.3 W m^−2^ was especially obtained with FKS-20/FAS-20 at *κ*_LS_ = 2.24 mS cm^−1^. In the past, 1.2 W m^−2^ has been reported with the RED stack having a comparable effective membrane area (100 cm^2^ × 25 FKD/FAD pairs = 5000 cm^2^ and 200 μm inter-membrane distance) using 30 g NaCl L^−1^ (≈0.51 M NaCl) as HS and 1 g NaCl L^−1^ (≈0.017 M NaCl) as LS [37]. The value in this study, 1.3 W m^−2^, to our best knowledge, is the highest power density in the world at lab-scale RED stacks (effective membrane area of 5000~7000 cm^2^). This will be due to the lower membrane resistance of FKS-20/FAS-20 (0.93 Ω cm^2^ at CEM/AEM pair) compared to FKD/FAD (3.03 Ω cm^2^ [6] at CEM/AEM pair) in the previous study. Compared to CIMS/ACS-8T (0.77 W m^−2^), FKS-20/FAS-20 had 1.7 times higher *PD*_max_ at *κ*_LS_ = 2.24 mS cm^−1^.

When using model SW as HS and model RW as LS, the values of *V*_OC_ were lower than the theoretical values at all *κ*_LS_. This is due to the concentration polarization on the membrane surface in the RED stack. FKE-20/FAS-20 had the highest *V*_OC_ in all CEMs/AEMs used in this study, which indicates FKS-20/FAS-20 may have the lowest osmotic water flow. *R*_int_ was lowest with FKS-20/FAS-20. This is attributed to the low-area-resistance and large current flow. When using FKS-20/FAS-20, the highest *V*_OC_ and the lowest *R*_int_ produced the highest output.

### 3.2. Model RO Brine and Model RW

#### 3.2.1. Comparison of the Open Circuit Voltage

**Figure 3:**
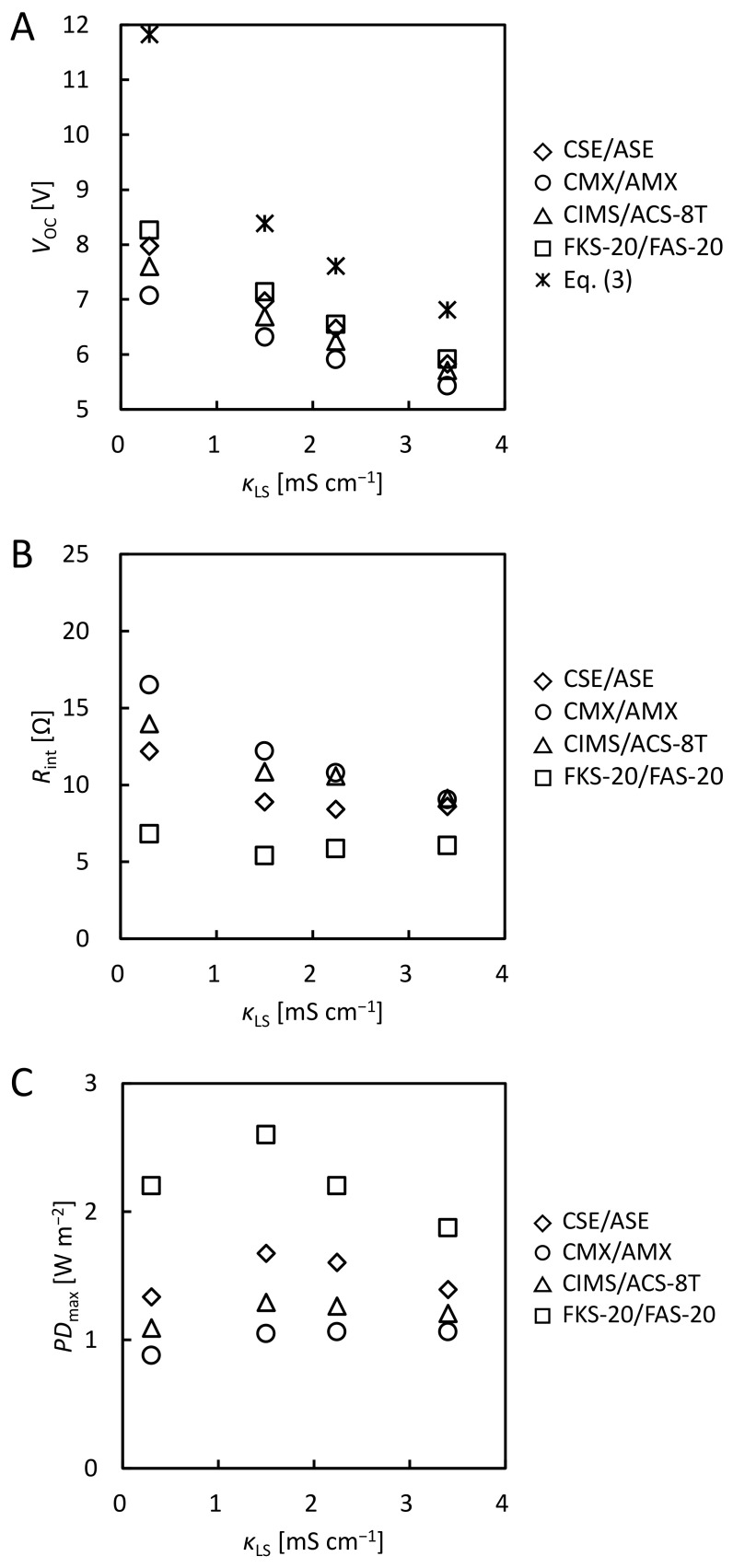
*κ*_LS_ dependence of (**A**) *V*_OC_, (**B**) *R*_int_, and (**C**) *PD*_max_ using model RO brine (90 mS cm^−1^ NaCl) as HS and model RW as LS.

Figure S5 shows the *V*-*I* curves of the RED stacks using the model RO brine and model RW with various membrane pairs. Figure 3A shows the *κ*_LS_ dependence of *V*_OC_ obtained from the *V*-*I* curves. For all IEM pairs, *V*_OC_ decreased as *κ*_LS_ increased owing to a decrease in *c*_HS_/*c*_LS_ (see Equation (3)). For all IEM pairs, because *c*_HS_/*c*_LS_ of model RO brine was larger than that of model SW, the *V*_OC_ for model RO brine was larger than that for model SW. *V*_OC_ increased in the following IEM pairs order: CMX/AMX < CIMS/ACS-8T < CSE/ASE < FKS-20/FAS-20, which is of the same order as *V*_OC_ using model SW (Figure 2A). The *V*_OC_ per pair of CIMS/ACS-8T was 0.19, 0.17, 0.16, and 0.14 V at *κ*_LS_ = 0.30, 1.50, 2.24, and 3.40 mS cm^−1^, respectively. In a previous study, the *V*_OC_ per pair of CIMS/ACS-8T using a pilot-scale RED stack at various feed flow rates was 0.17~0.18 V using 90 mS cm^−1^ NaCl as HS and 0.34 mS cm^−1^ surface water as LS [27]. This indicates that not only the use of model SW but also model RO brine as HS, the *V*_OC_ per pair of CIMS/ACS-8T using the lab-scale RED stack in this study showed almost the same values as those using the pilot-scale RED stack. *V*_OC_ obtained from the experiment in all IEM pairs was 20% to 40% lower than *V*_OC, theory_ calculated from Equation (3) (11.8, 8.4, 7.6, and 6.8 V).

To examine the cause of the differences in *V*_OC_ between IEM pairs and the deviation between *V*_OC_ and *V*_OC,theory_, the membrane potential of a diffusion dialysis system consisting of an IEM, and 90 and 1.50 mS cm^−1^ NaCl solutions was measured and is shown in Figure S6A. The absolute values of the membrane potential were approximately 102 mV for all the CEMs and 91 mV for all the AEMs. The membrane potential generated when 40 pairs of CEMs/AEMs were stacked (total *ϕ*_m_) is shown in Figure S6B. The calculated values of all IEM pairs (≈7.7 V) were lower than the ideal membrane potential (8.4 V) calculated using Equation (3). This could be attributed to a decrease in the transport number of IEMs owing to the use of a high concentration of NaCl solution (≈1.2 M), and the concentration ratio at the two membrane surfaces will decrease because of the external concentration polarization caused by osmotic water flow. CIMS/ACS-8T had the highest total *ϕ*_m_ in all IEM pairs; however, *V*_OC_ was lower than that of CSE/ASE and FKS-20/FAS-20. The difference between the *V*_OC_ of the RED stack and the total *ϕ*_m_ in the diffusion dialysis system will be due to the difference in the concentration polarization between the RED stack [27] and the diffusion dialysis system mentioned in Section 3.1.1.

#### 3.2.2. Comparison of the Internal Resistance

Figure 3B shows the *κ*_LS_ dependence of *R*_int_ obtained from the slope of the *V*-*I* curve (Figure S5) at the current when *P*_max_ was obtained. *R*_int_ decreased with *κ*_LS_ due to a decrease in the conductivity of the LS side in the RED stack. *R*_int_ increased in the following IEM pairs order: FKS-20/FAS-20 < CSE/ASE < CIMS/ACS-8T < CMX/AMX. *R*_int_ of FKS-20/FAS-20 were 5.4~6.8 Ω and the values were 30~59% lower than the other IEM pairs. This is attributed to the lowest area resistance of FKS-20/FAS-20 in all the IEM pairs and the increase in the conductivity of the LS channel due to the high current (see Figure S5). Additionally, the *κ*_LS_ dependence of *R*_int_ of FKS-20/FAS-20 showed strange trends that were different from other IEM pairs, that is, *R*_int_ increased with the increase of *κ*_LS_ at *κ*_LS_ ≥ 1.5 mS cm^−1^. This will be due to the following reason: *R*_int_ was calculated at the current when *P*_max_ was obtained. At *κ*_LS_ = 1.5 mS cm^−1^, FKS-20/FAS-20 showed the current value of 0.58 A at *P*_max_, while the current values were about 0.52 A and 0.47 A at *P*_max_ at *κ*_LS_ = 2.24 and 3.40 mS cm^−1^, respectively. Thus, the high current will decrease the resistance of LS channel. Therefore, FKS-20/FAS-20 showed the lowest *R*_int_ at *κ*_LS_ = 1.5 mS cm^−1^. All IEM pairs except for FKS-20/FAS-20 had almost the same *R*_int_ value for each other at *κ*_LS_ = 3.40 mS cm^−1^. This is because at the high conductivities, the contribution of the resistance of HS and LS channels on *R*_int_ will be larger than that of the membrane resistance. Except for the CMX/AMX at *κ*_LS_ = 1.50, 2.24, and 3.40 mS cm^−1^, *R*_int_ values in all the IEM pairs were lower than those with model SW. Model RO brine has lower resistance than model SW, and the increase in *κ*_LS_ caused by ion diffusion from the HS channel will be greater than from model SW due to the concentration difference, resulting in above lower *R*_int_ values. When model SW was used in CMX/AMX, internal leakage in the RED stack may have occurred, which caused an increase in *κ*_LS_ and a decrease in *R*_int_.

The *κ*_LS_ dependence of the calculated values (*R*_int,cal_) is shown in Figure S7. At *κ*_LS_ = 0.30 mS cm^−1^, *R*_int,cal_ is large (≈60 Ω) because of the low conductivity of the LS channel. For the same reason as the model SW, the experimental values were much lower than *R*_int,cal_ at *κ*_LS_ = 0.30 mS cm^−1^. At *κ*_LS_ ≥ 1.50 mS cm^−1^, the *R*_int,cal_ values were not much different from those of model SW because the contribution of the *R*_HS_ to *R*_int,cal_ was low. However, the experimental values (*R*_int_) using the model RO brine were lower than the values of *R*_int,cal_ because of the increase in the conductivity of the LS channel caused by ion diffusion from the HS channel.

#### 3.2.3. Comparison of the Maximum Power Density

Figure 3C shows the *κ*_LS_ dependence of the *PD*_max_. For all IEM pairs, *PD*_max_ reached maximum values around *κ*_LS_ = 1.50 and 2.24 mS cm^−1^. Owing to an increase in the concentration ratio of HS and LS, the maximum *PD*_max_ with the model RO brine was larger than that with the model SW for all IEM pairs. *PD*_max_ increased in the following IEM pairs order: CMX/AMX < CIMS/ACS-8T < CSE/ASE < FKS-20/FAS-20. FKS-20/FAS-20 showed maximum *PD*_max_ (2.6 W m^−2^) at *κ*_LS_ = 1.50 mS cm^−1^ because of the lowest area resistances in all the IEM pairs (see Table 1). Additionally, compared to CIMS/ACS-8T (1.3 W m^−2^), FKS-20/FAS-20 showed 2.0 times larger *PD*_max_ at *κ*_LS_ = 1.50 mS cm^−1^. In the case of model SW, CMX/AMX showed the second highest *PD*_max_; however, in the case of the model RO brine, they had the lowest *PD*_max_. This is due to the lowest *V*_OC_ and highest *R*_int_ of the IEM pair.

When using model RO brine as HS and model RW as LS, the values of *V*_OC_ were lower than the values of *V*_OC,theory_, and the difference between *V*_OC_ and *V*_OC,theory_ was larger than when model SW was used as HS. This is due to the large difference in concentration between HS and LS, resulting in a large osmotic water flow and a large concentration polarization at the membrane surface. FKE-20/FAS-20 area resistance is low, showing the largest *V*_OC_ and the lowest *R*_int_ in all CEMs/AEMs, and produced the highest output.

### 3.3. Natural SW and Surface Water

#### 3.3.1. Comparison of the Open Circuit Voltage

**Figure 4:**
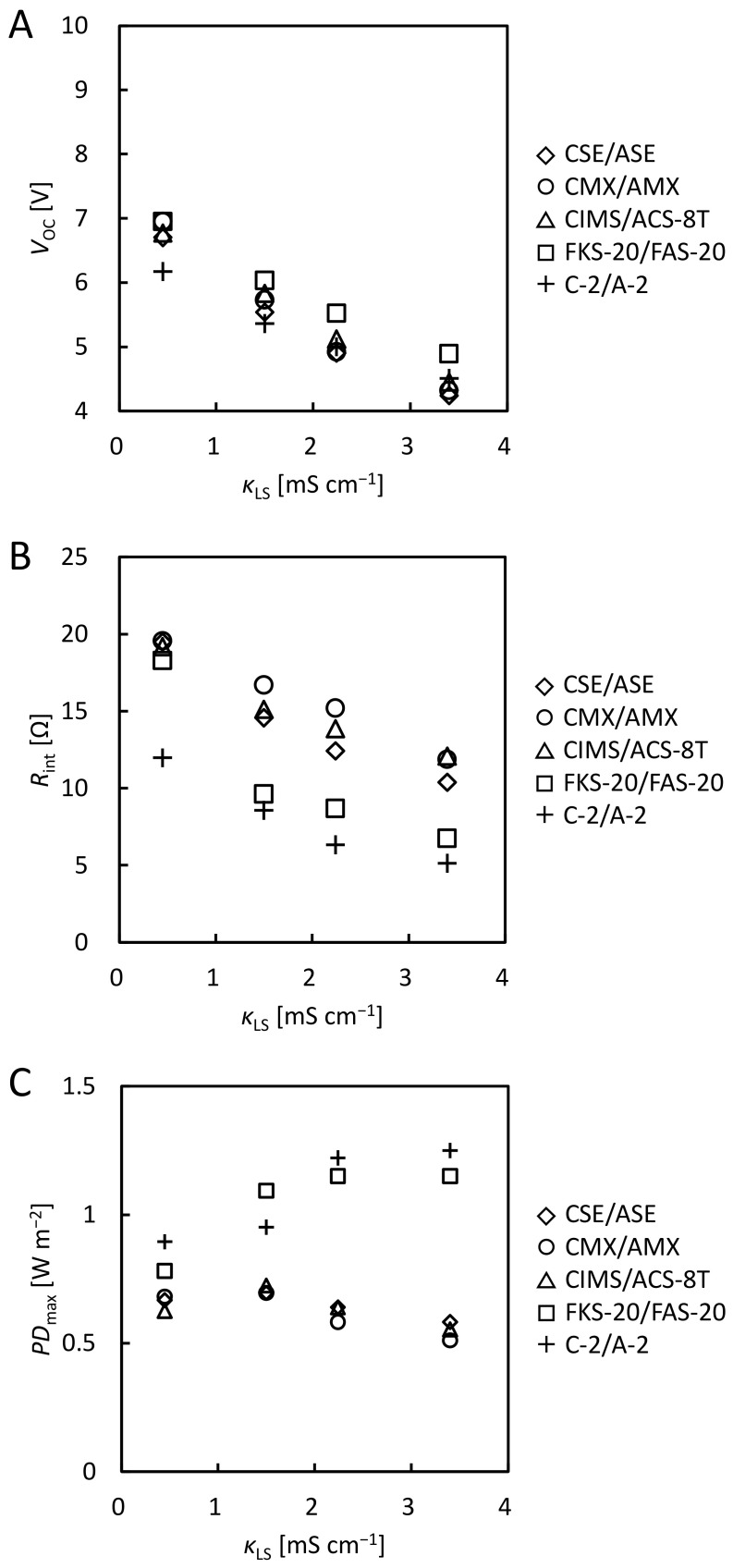
*κ*_LS_ dependence of (**A**) *V*_OC_, (B) *R*_int_, and (C) *PD*_max_ using natural SW as HS and surface water as LS.

Figure S8 shows the *V*-*I* curves of the RED stacks using natural SW and surface water with various membranes. Figure 4A shows the *κ*_LS_ dependence of *V*_OC_ obtained from the *V*-*I* curves. For all IEM pairs, *V*_OC_ decreased as *κ*_LS_ increased owing to a decrease in *c*_HS_/*c*_LS_ (see Equation (3)). The values using natural SW were 1~14% smaller than those using model SW. Natural SW contains K^+^, Mg^2+^, Ca^2+^, and SO_4_^2−^, in addition to Na^+^ and Cl^−^ contained in the model SW. In particular, the presence of divalent ions causes a decrease in *V*_OC_ [11,18,19,20,38,39]. Although the order of *V*_OC_ for the IEM pairs varied with the value of *κ*_LS_, FKS-20/FAS-20 had the highest *V*_OC_ (7.0, 6.0, 5.5, and 4.9 V) at all *κ*_LS_ value. This is the same as for the model SW and the model RO brine, which may be ascribed to the low osmotic water flow. *V*_OC_ of the other IEMs pairs differed little (about 6.8, 5.6, 5.0, and 4.4 V) except for C-2/A-2 at *κ*_LS_ = 0.45 and 1.50 mS cm^−1^. C-2/A-2 is thinner than other IEMs (see Table 1) and may cause high osmotic water flow, which caused a concentration polarization and the low *V*_OC_. Almost equal *V*_OC_ of CMX/AMX and CIMS/ACS-8T is consistent with the result that there was little difference in *V*_OC_ when natural SW and distilled water were supplied to the RED stack (10 membrane pairs, 200 μm inter-membrane distance, 1760 cm^2^ total effective membrane area) using CMX/AMX and CIMS/ACS-8T [29]. *V*_OC_ per pair of CIMS/ACS-8T using the lab-scale stack was 0.17, 0.15, 0.13, and 0.11 V at *κ*_LS_ = 0.45, 1.5, 2.2, and 3.4 mS cm^−1^, respectively. In a previous study, *V*_OC_ per CIMS/ACS-8T pair using a pilot-scale RED stack at various feed flow rates was 0.15~0.17 V using 52 mS cm^−1^ natural SW as HS and 0.34 mS cm^−1^ surface water as LS [27].

#### 3.3.2. Comparison of the Internal Resistance

Figure 4B shows the *κ*_LS_ dependence of *R*_int_ calculated from the slope of the *V*-*I* curve (Figure S8) at the current when *P*_max_ was obtained. For all IEMs pairs, *R*_int_ decreased as *κ*_LS_ increased because the resistance of LS decreased. Except for *κ*_LS_ = 0.45 mS cm^−1^, *R*_int_ values were about 10% larger for CSE/ASE, CIMS/ACS-8T, and FKS-20/FAS-20 and 45% larger for CMX/AMX than those using model SW. This is due to the lower molar mobility of divalent ions in a membrane compared to monovalent ions such as Na^+^ and Cl^−^, resulting in a larger *R*_int_ using natural SW containing the divalent ions [18,38,40]. C-2/A-2 had the lowest *R*_int_ (12, 8.6, 6.3, and 5.1 Ω) in all IEM pairs, with FKS-20/FAS-20 and CMX/AMX having the second lowest and the highest *R*_int_, respectively. This result is ascribed to the lowest area resistance of C-2/A-2 (see Table 1) and high current value at *P*_max_ (see Figure S8).

The *κ*_LS_ dependence of *R*_int,cal_ is shown in Figure S9. Because the calculations do not consider the presence of multivalent ions, the values of *R*_int,cal_ in natural SW were almost the same as those in Model SW. As in the case of model SW and model RO brine, the experimental values at *κ*_LS_ = 0.45 mS cm^−1^ were lower than *R*_int,cal_ values in all the IEM pairs. The experimental values using CSE/ASE and CIMS/ACS-8T at *κ*_LS_ ≥ 2.24 mS cm^−1^ and using CMX/AMX at *κ*_LS_ ≥ 1.50 mS cm^−1^ were larger than *R*_int,cal_, probably because of the effect of multivalent ions. In the case of FKS-20/FA-20 and C-2/A-2, the experimental values at *κ*_LS_ ≥ 1.50 mS cm^−1^ were larger than *R*_int,cal_ because the decrease in the conductivity of the LS channel caused by the high current (see Figure S8) may be larger than the effect of multivalent ions.

#### 3.3.3. Comparison of the Maximum Power Density

Figure 4C shows the *κ*_LS_ dependence of the *PD*_max_. CSE/ASE, CMX/AMX, and CIMS/ACS-8T had almost the same *PD*_max_ as each other (about 0.66, 0.71, 0.62, 0.55 W m^−2^ at each *κ*_LS_). For FKS-20/FAS-20 and C-2/A-2, *PD*_max_ increased as *κ*_LS_ increased (from 0.78 to 1.3 W m^−2^). For all IEM pairs, the values of *PD*_max_ using natural SW were 4~39% smaller than those using model SW because of the smaller *V*_OC_ and larger *R*_int_. The decrease in *PD*_max_ due to the use of a natural SW has been reported [16,17,19,38]. *PD*_max_ was largest when C-2/A-2 was used because it had the lowest *R*_int_ among all IEM pairs (see Figure 4B) except for *κ*_LS_ = 1.50 mS cm^−1^. In particular, 1.3 W m^−2^ was obtained at *κ*_LS_ = 3.40 mS cm^−1^, and this value was 2.3 times higher than that with CIMS/ACS-8T (0.55 W m^−2^).

When using natural SW as HS and surface water as LS, *V*_OC_ was lower and *R*_int_ was higher than when model SW was used, which is due to the presence of multivalent ions. Although C-2/A-2 did not show the highest *V*_OC_, C-2/A-2 showed the lowest *R*_int_ due to the lowest area resistance, and thus produced the largest output in all IEM pairs.

### 3.4. Prediction of the Power Output with a Pilot-Scale RED Stack

Table 3 shows a summary of the best performance of the lab-scale RED stack in this study. *V*_OC_ per one FKS-20/FAS-20 pair was 5.7/40 = 0.143 V and 7.1/40 = 0.178 V when using model SW and model RO brine, and *V*_OC_ per one C-2/A-2 pair was 4.5/40 = 0.113 V when using natural SW. Compared to CIMS/ACS-8T as a control, FKS-20/FAS-20 showed 1.7 and 2.0 times higher *PD*_max_ when using model SW and model RO brine, and C-2/A-2 showed 2.3 times higher *PD*_max_ when using natural SW. We evaluated the performance of the pilot-scale RED stack with CIMS/ACS-8T (299 membrane pairs, 200 μm inter-membrane distance, 179.4 m^2^ total effective membrane area) using model SW (50 mS cm^−1^ NaCl), model RO brine (90 mS cm^−1^ NaCl), and natural SW as HS and surface water (the value of conductivity was 0.34 mS cm^−1^) as LS, and obtained a power output of 174 W, 263 W and 111 W, respectively [27]. In the pilot-scale RED stack, the flow rate was set to 21 L min^−1^ of HS and 32 L min^−1^ of LS to obtain above power outputs. The pumping energies at the flow rate were 44 W with model SW, 41 W with model RO brine, and 31 W with natural SW, respectively. When we assume that osmotic water flow through the IEMs and the internal salt leakage are comparable between the lab and the pilot-scale RED stacks, *V*_OC_ in the pilot-scale RED stack in a case of using FKS-20/FAS/20 and C-2/A-2 using model SW, model RO brine, and natural SW are expected to be 0.143 × 299 ≈ 43 V, 0.178 × 299 ≈ 53 V, and 0.113 × 299 ≈ 34 V, respectively based on a proportional calculation of the number of IEM pair. Assuming that the SGE conversion efficiency is equal for the lab and the pilot-scale RED stack and using the *PD*_max_ ratio to CIMS/ACS-8T, power outputs of 174 × 1.7 ≈ 300 W and 263 × 2.0 ≈ 530 W can be expected in the pilot-scale RED stack with the FKS-20/FAS-20 using model SW and model RO brine at the flow rate of 21 L min^−1^ of HS and 32 L min^−1^ of LS. When using natural SW, the pilot-scale RED stack with C-2/A-2 can produce 111 × 2.3 ≈ 260 W. Net power outputs calculated by subtracting the pumping energy were 256 W using model SW, 489 W using model RO brine, and 229 W using natural SW. It is possible that residence time of the solution in the pilot-scale RED stack will increase, and the osmotic water flow will become high, resulting in a slightly lower power output than the prediction. However, it may be possible to achieve the output predicted in this study by trying other methods of increasing RED output, such as using profiled membranes [41,42,43,44,45].

## 4. Conclusions

In this study, we clarified IEM properties that are effective for RED power generation. In addition, we predicted that the pilot-scale RED stack can produce large outputs by using appropriate IMEs. The key findings are described below.

IEMs with low-area-resistance, such as FKS-20/FAS-20 and C-2/A-2, produced high power outputs in all feed solutions due to the high current.

The monovalent selective membranes (CIMS/ACS-8T) did not contribute to an increase in the power output, though they may be effective in preventing IEM fouling.

For RED power generation, IEMs that can generate a high current flow even if *V*_OC_ slightly decreases due to osmotic water flow will be effective. Of course, IEMs with low-area-resistance and low osmotic water flow would be able to produce higher power output.

Using appropriate IEMs in the pilot-scale RED stack, we predicted 1.7~2.3 times the power output of the previous study. Although these values are only conjectures, they can be indicators of the power output of large RED stacks.

## Figures and Tables

**Table 1:** Basic properties of IEMs used in this study.

Membrane		Charge Density [M]	Ion Transport Number [-]	Area Resistance [Ω cm^2^]	Thickness [μm]
CEM	CSE	1.90	0.98	2.03	150
	CMX	1.86	0.98	2.70	170
	CIMS	-	0.98	2.49	150
	C-2	-	0.94	0.21	34
	FKS-20	1.93	0.98	0.47	18
AEM	ASE	1.84	0.98	2.77	150
	AMX	1.53	0.98	2.30	140
	ACS-8T	-	0.98	2.41	150
	A-2	-	0.99	0.28	34
	FAS-20	2.14	0.97	0.46	23

**Table 2:** Ion composition in natural SW and surface water.

Solution	Cations [mM]				Anions [mM]		TDS, ppm *
	Na^+^	K^+^	Mg^2+^	Ca^2+^	Cl^−^	SO_4_^2−^	
Natural SW (55.2~55.4 mS cm^−1^)	471	29	62	15	503	25	34300
Surface water (0.43~0.45 mS cm^−1^)	0.89	0.19	0.19	0.92	0.97	0.20	123

* TDS (total dissolved sloid) was calculated by the ion composition, and we assumed mg L^−1^ equals ppm.

**Table 3:** Summary of the best performance of the lab-scale RED stack in this study (40 membranes pairs, 200 μm inter-membrane distance, and 7040 cm^2^ total effective membrane area).

Feed Solutions	CEM/AEM Pair	*V*_OC_ [V]	*R*_int_ [Ω]	*PD*_max_ [W m^−2^]	*PD*_max_ Ratio to CIMS/ACS-8T
Model SW and model RW (*κ*_LS_ = 2.24 mS cm^−1^)	FKS-20/FAS-20	5.7	7.6	1.3	1.7
Model RO brine and model RW (*κ*_LS_ = 1.50 mS cm^−1^)	FKS-20/FAS-20	7.1	5.4	2.6	2.0
Natural SW and surface water (*κ*_LS_ = 3.40 mS cm^−1^)	C-2/A-2	4.5	5.1	1.3	2.3

## Data Availability

The data that support the findings of this study are available from the corresponding author, M.H., upon reasonable request.

## Supplementary Materials

The following supporting information can be downloaded at: https://www.mdpi.com/article/10.3390/membranes12111141/s1, Figure S1: Flow diagram of the performance evaluation of the RED stack; Table S1: Sodium chloride activity coefficient at the molar concentrations; Figure S2: *V*-*I* and *P*-*I* curves using model SW (50 mS cm^−1^ NaCl) as HS and model RW as LS; Figure S3: *ϕ*_m_ measured in 50 mS cm^−1^ and 1.50 mS cm^−1^ NaCl solutions combination; Figure S4: *κ*_LS_ dependence of *R*_int,cal_ using model SW (50 mS cm^−1^ NaCl) as HS and model RW as LS; Figure S5: *V*-*I* and *P*-*I* curves using model RO brine (90 mS cm^−1^ NaCl) as HS and model RW as LS; Figure S6: *ϕ*_m_ measured in 90 mS cm^−1^ and 1.50 mS cm^−1^ NaCl solutions combination; Figure S7: *κ*_LS_ dependence of *R*_int,cal_ using model RO brine (90 mS cm^−1^ NaCl) as HS and model RW as LS; Figure S8: *V*-*I* and *P*-*I* curves using natural SW as HS and surface water as LS; Figure S9: *κ*_LS_ dependence of *R*_int,cal_ using natural SW as HS and surface water as LS; Figure S10: Set up of electrode resistance measurement; Figure S11: *V*-*I* curves of the stack without IEMs before and after a series of power generation test using model SW and model RW; Figure S12: *V*-*I* curves of the stack without IEMs before and after a series of power generation test using model RO brine and model RW; Figure S13: *V*-*I* curves of the stack without IEMs before and after a series of power generation test using natural SW and surface water; Figure S14: Spacer net geometry. References [36,46] are cited in the Supplementary Materials.
