# Supplementary material for: Power Generation Performance of Reverse Electrodialysis (RED) Using Various Ion Exchange Membranes and Power Output Prediction for a Large RED Stack
Source: Membranes (Basel). 2022 Nov 13;12(11):1141. doi: 10.3390/membranes12111141 (PMC9697558; doi:10.3390/membranes12111141)
Supplement: Supplementary file 1 [file membranes-12-01141-s001.zip › membranes-2005546-supplementary.pdf]

Supplementary materials

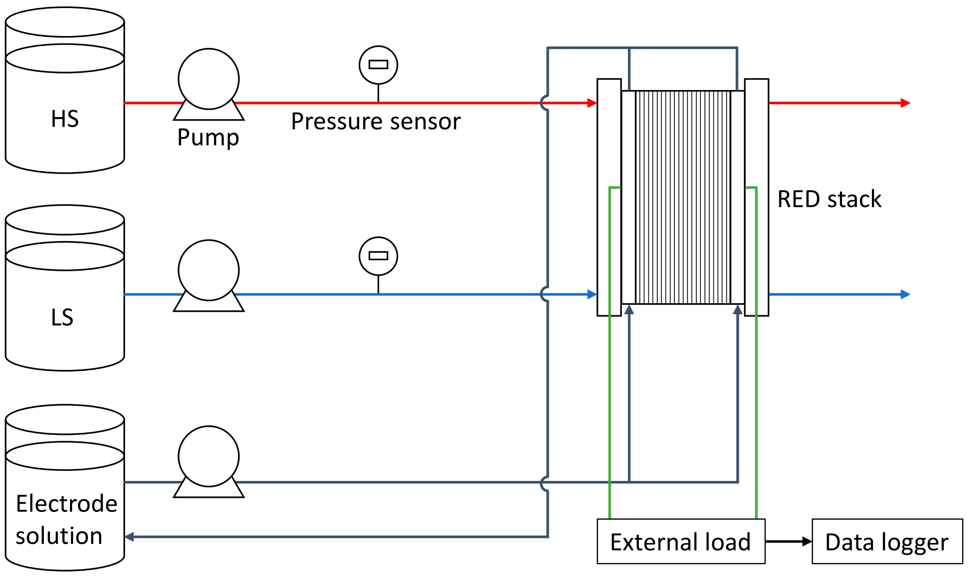

Figure S1. Flow diagram of the performance evaluation of RED stack.

Table S1. Sodium chloride activity coefficient at the molar concentrations [1].

c_{NaCl} [mM]	γ_{NaCl}
3.7	0.936
13.5	0.891
20.3	0.871
31.1	0.846
516	0.679
1180	0.655

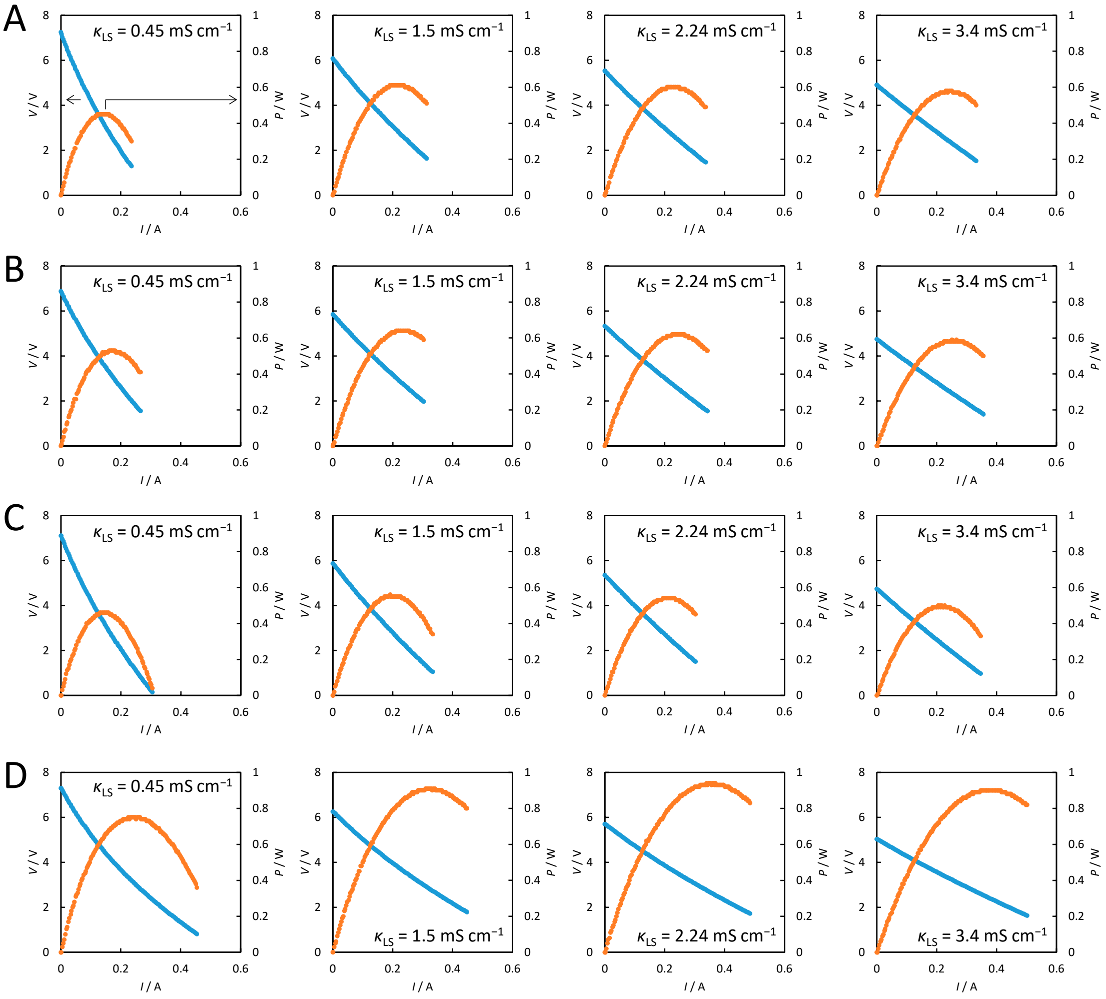

Figure S2. *V-I* and *P-I* curves using model SW (50 mS cm^{-1} NaCl) as HS and model RW as LS with (A) CSE/ASE, (B) CMX/AMX (C) CIMS/ACS-8T, and (D) FKS-20/FAS-20.

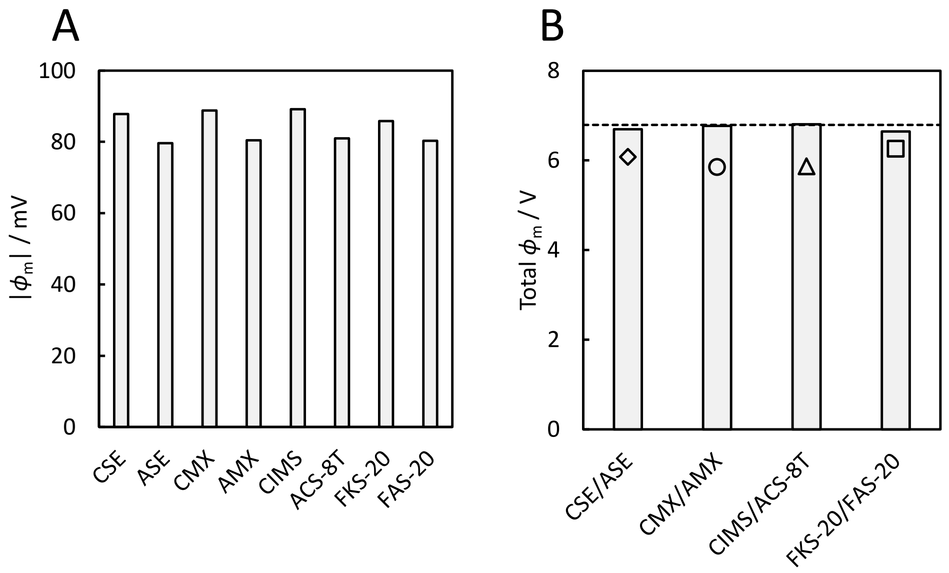

Figure S3. (A) ϕ_m measured in 50 mS cm^{-1} and 1.50 mS cm^{-1} NaCl solutions combination. (B) Total ϕ_m with 40 pairs of CEM/AEM. The dashed line shows the theoretical value calculated from Eq. (3). The symbols show V_{OC} values of the RED stack using model SW at κ_{LS} = 1.50 mS cm^{-1}.

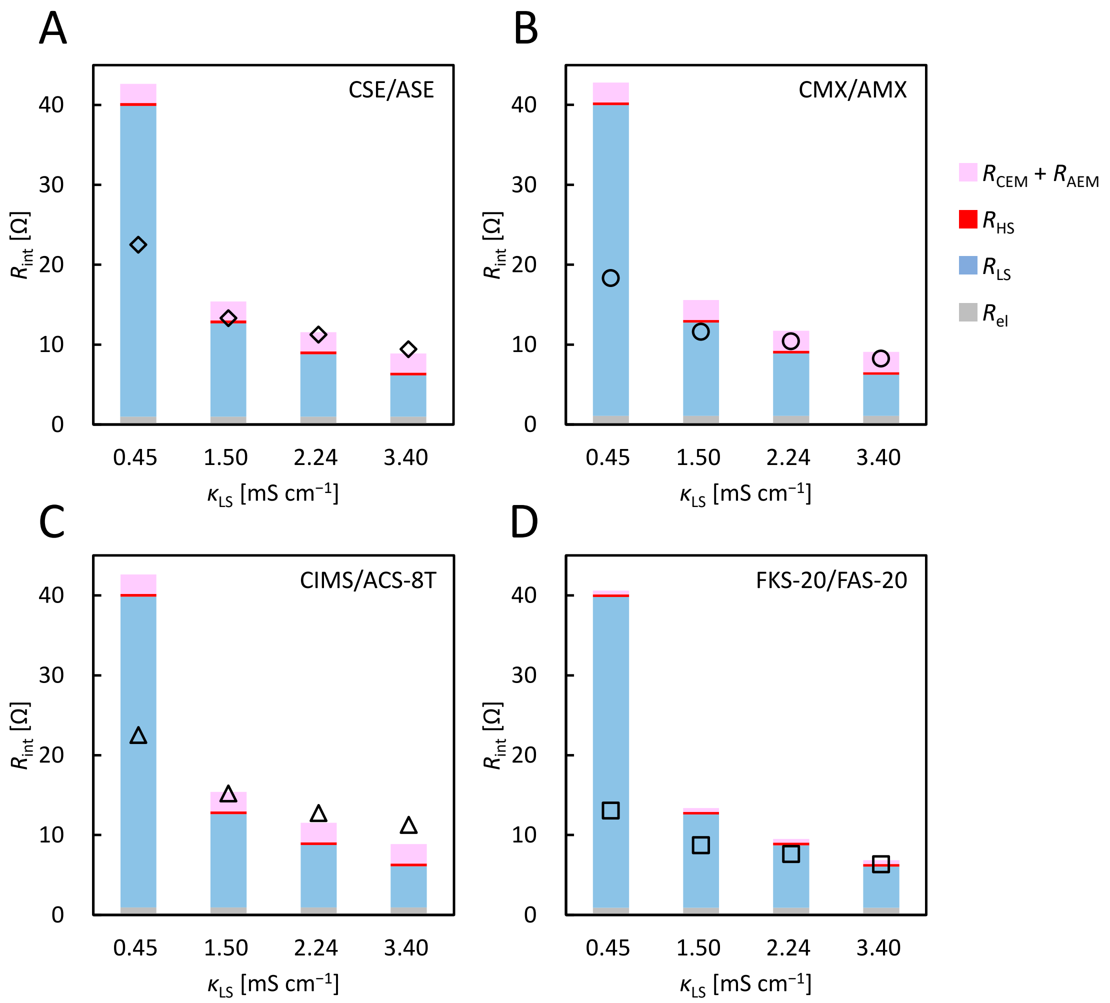

Figure S4. κ_{LS} dependence of $R_{int,cal}$ using model SW (50 mS cm^{-1} NaCl) as HS and model RW as LS with (A) CSE/ASE, (B) CMX/AMX (C) CIMS/ACS-8T, and (D) FKS-20/FAS-20. R_{el} (gray) are the experimental values obtained from the *V*-*I* curves shown in Figure S11. R_{HS} and R_{LS} are calculated from Eqs. (9) and (10) using the conductivities of HS and LS, R_{CEM} and R_{AEM} are calculated from Eqs. (11) and (12) using values of the area resistance in Table 1. The symbols show experimental values.

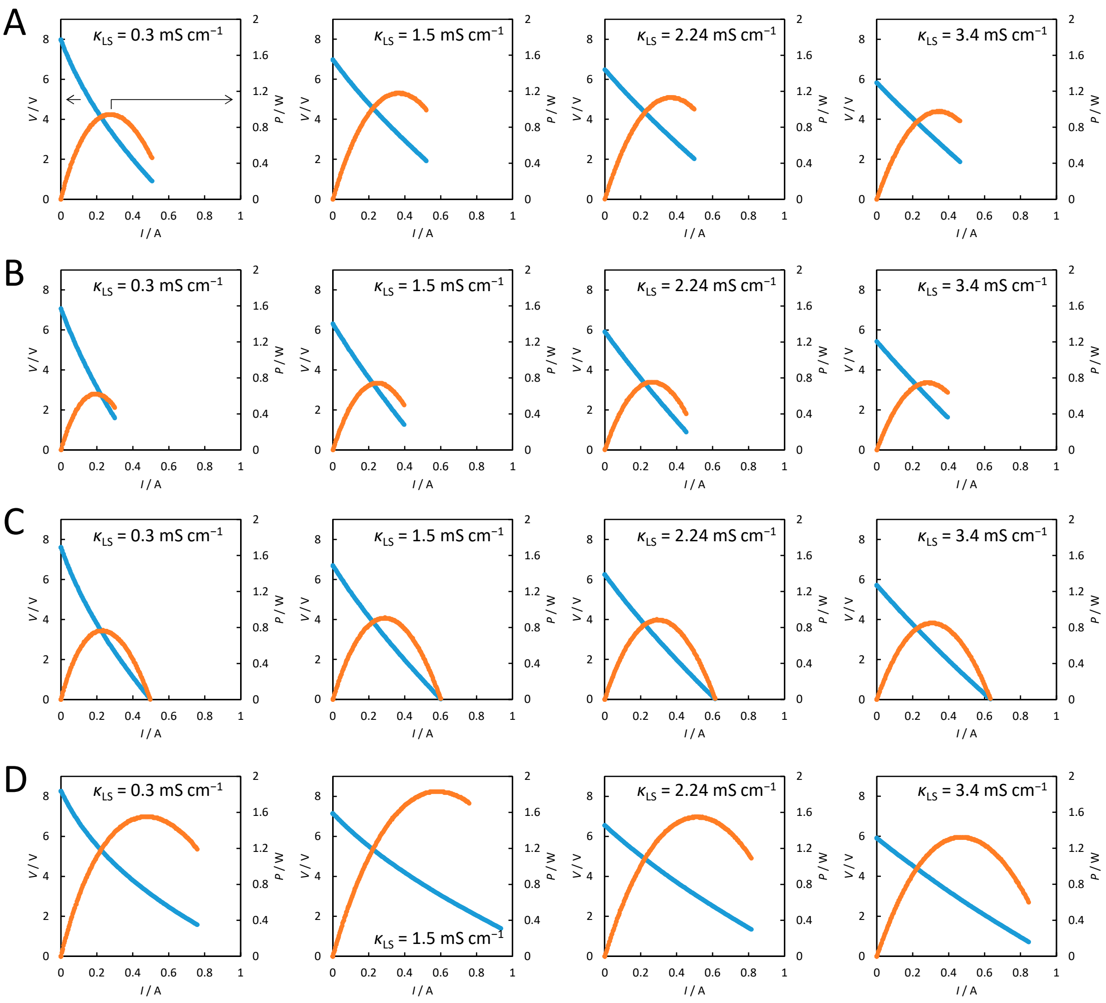

Figure S5. *V*-*I* and *P*-*I* curves using model RO brine (90 mS cm^{-1} NaCl) as HS and model RW as LS with (A) CSE/ASE, (B) CMX/AMX (C) CIMS/ACS-8T, and (D) FKS-20/FAS-20.

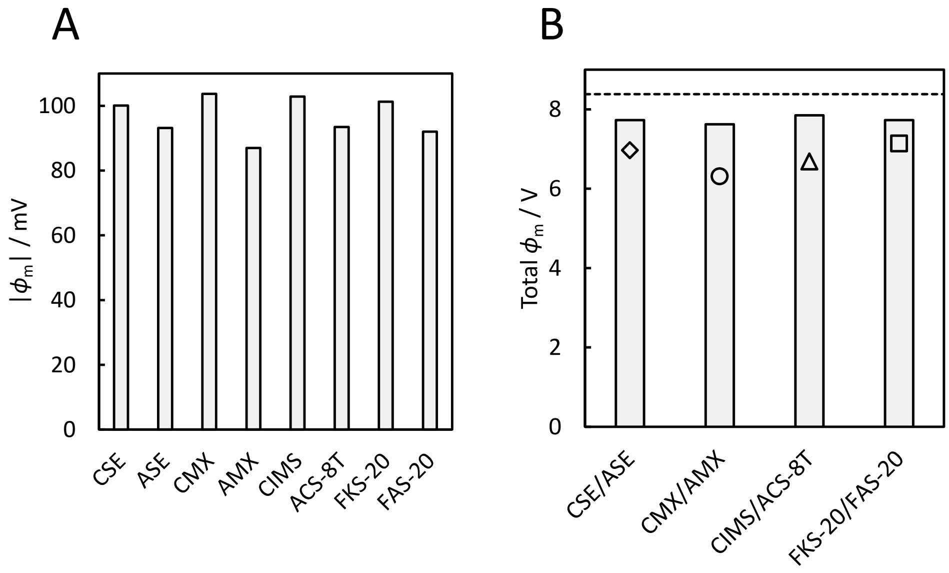

Figure S6. (A) ϕ_m measured in 90 mS cm^{-1} and 1.50 mS cm^{-1} NaCl solutions combination. (B) Total ϕ_m with 40 pairs of CEM/AEM. The dashed line shows the theoretical values calculated from Eq. (3). The symbols show V_{OC} values of the RED stack using model RO brine at κ_{LS} = 1.50 mS cm^{-1}.

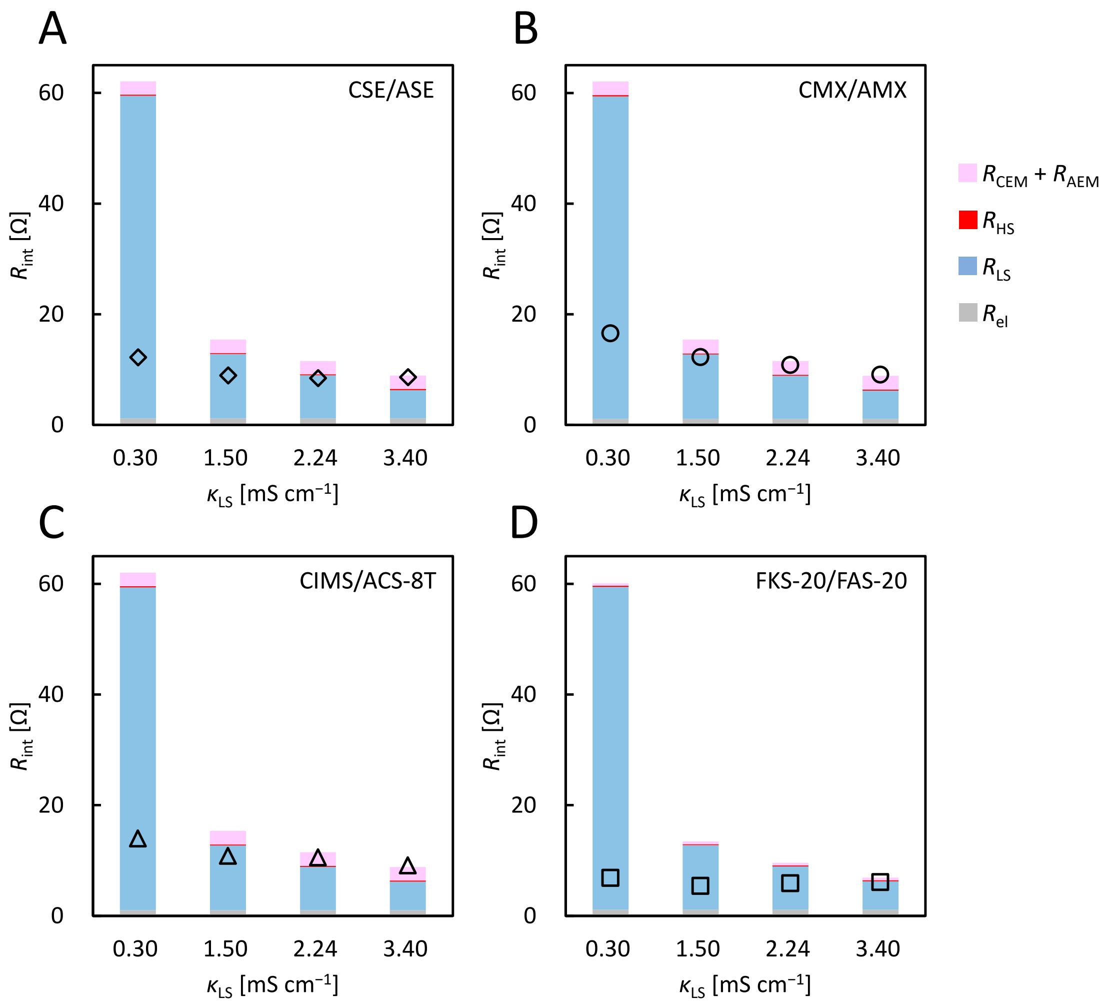

Figure S7. κ_{LS} dependence of $R_{int,cal}$ using Model RO brine (90 mS cm^{-1} NaCl) as HS and model RW as LS with (A) CSE/ASE, (B) CMX/AMX (C) CIMS/ACS-8T, and (D) FKS-20/FAS-20. R_{el} (gray) are the experimental values obtained from the *V-I* curves shown in Figure S12. R_{HS} and R_{LS} are calculated from Eqs. (9) and (10) using the conductivities of HS and LS, R_{CEM} and R_{AEM} are calculated from Eqs. (11) and (12) using values of the area resistance in Table 1. The symbols show experimental values.

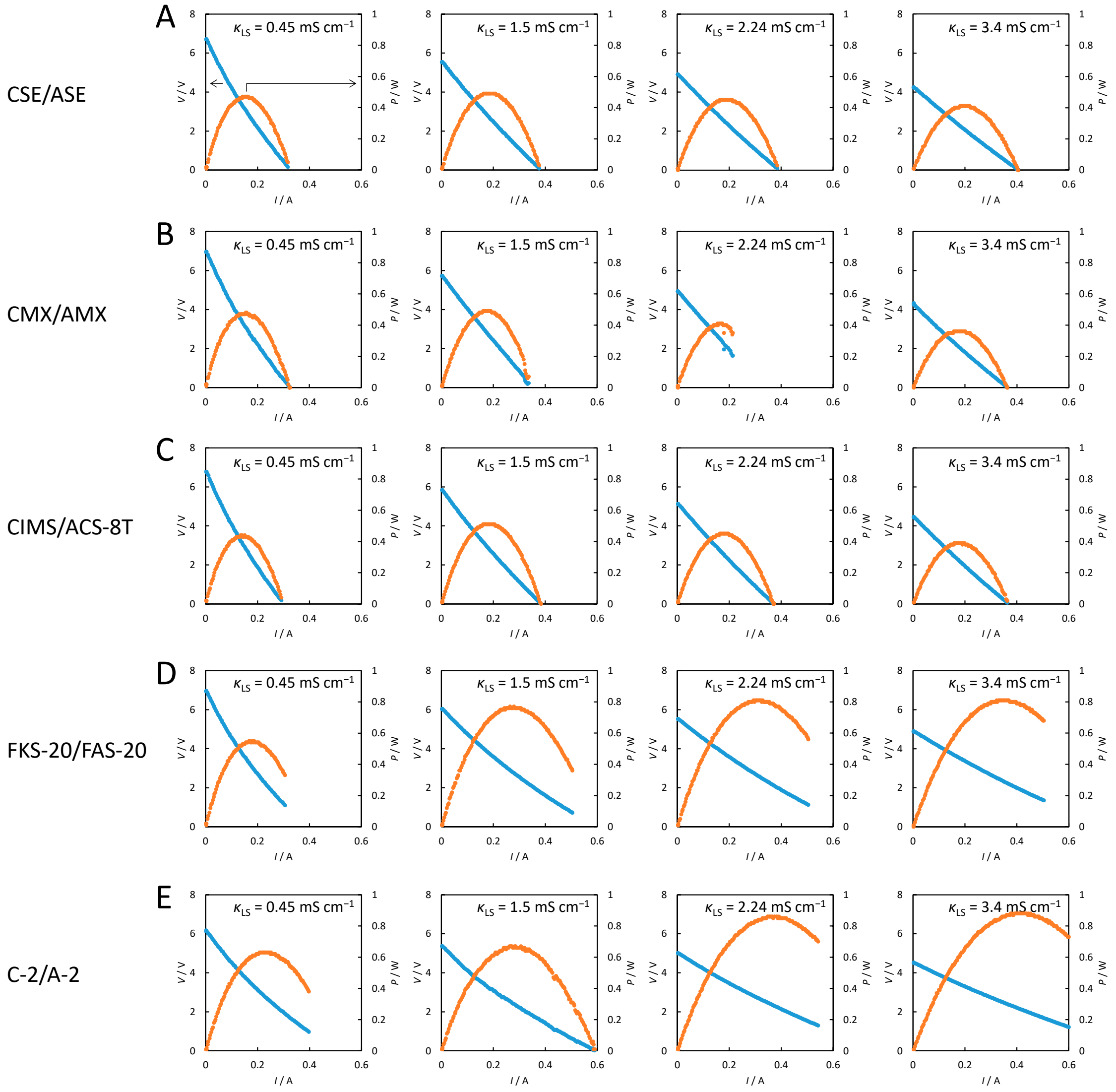

Figure S8. *V-I* and *P-I* curves using natural SW as HS and surface water as LS with (A) CSE/ASE, (B) CMX/AMX (C) CIMS/ACS-8T, (D) FKS-20/FAS-20, and (E) C-2/A-2.

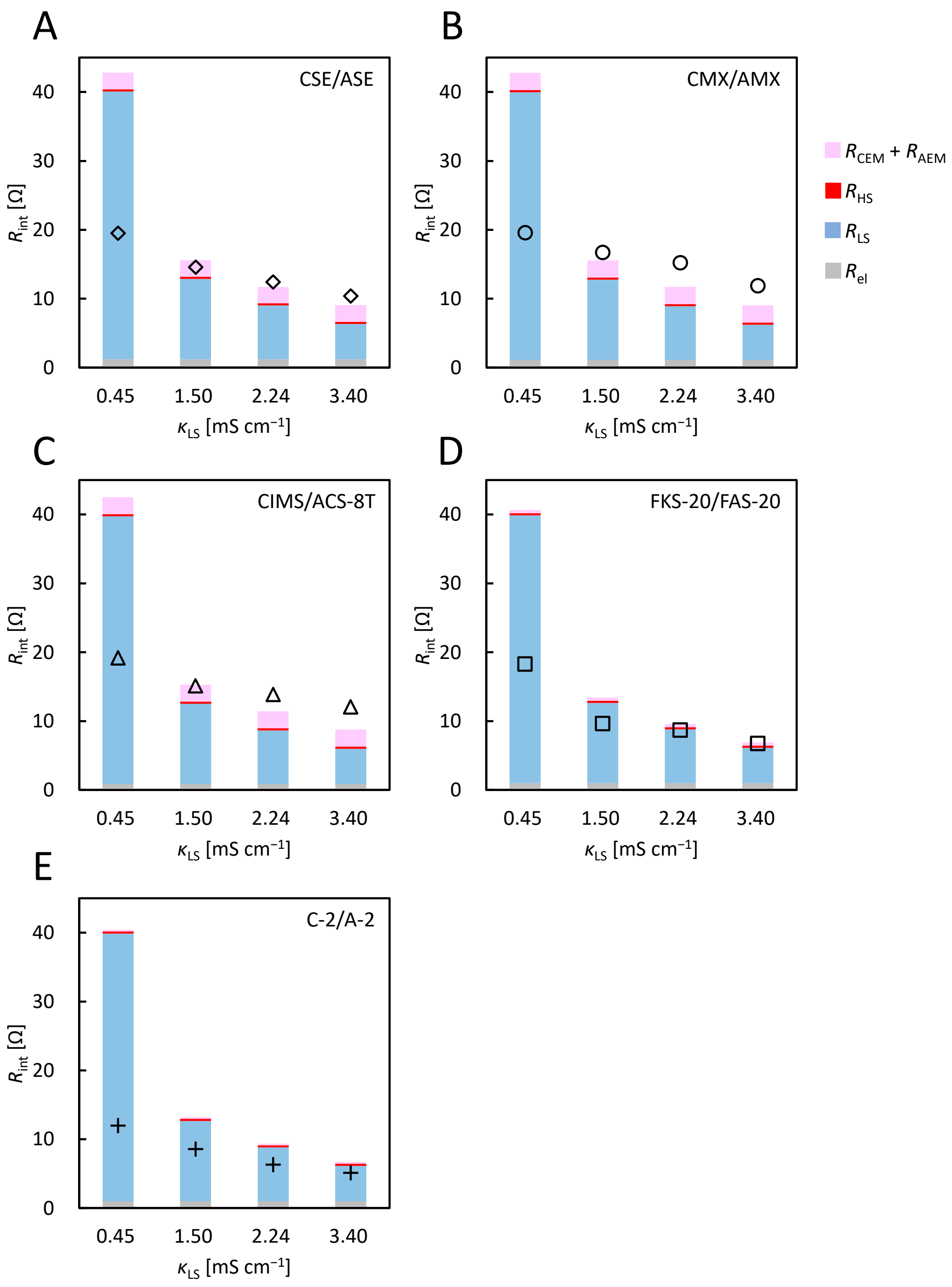

Figure S9. κ_{LS} dependence of $R_{int,cal}$ using natural SW as HS and surface water as LS with (A) CSE/ASE, (B) CMX/AMX (C) CIMS/ACS-8T, (D) FKS-20/FAS-20, and (E) C-2/A-2. R_{el} (gray) are the experimental values obtained from the *V-I* curves shown in Figure S13. R_{HS} and R_{LS} are calculated from Eqs. (9) and (10) using the conductivities of HS and LS, R_{CEM} and R_{AEM} are calculated from Eqs. (11) and (12) using values of the area resistance in Table 1. The symbols show the experimental values.

•Measurement of R_{el}

Before and after a series of power generation test of the RED stack with each CEM/AEM (V-I curve measurements at the all conductivities of LS), 3.0 M NaCl solution was supplied to the stack without IEMs (Figure S10). DC power supply was operated in constant voltage mode with the voltage value of 6.0 V, which is close to the RED stack V_{OC} in this study. I was varied from 0 A with the changing rate of 0.2 A min^{-1} to measure V-I curve. The average of the absolute values of slope of the two V-I curves before and after the series of power generation test was taken as R_{el}. R_{el} ranged from 0.85 to 1.3 Ω in all experiments (Figure S11, S12, and S13).

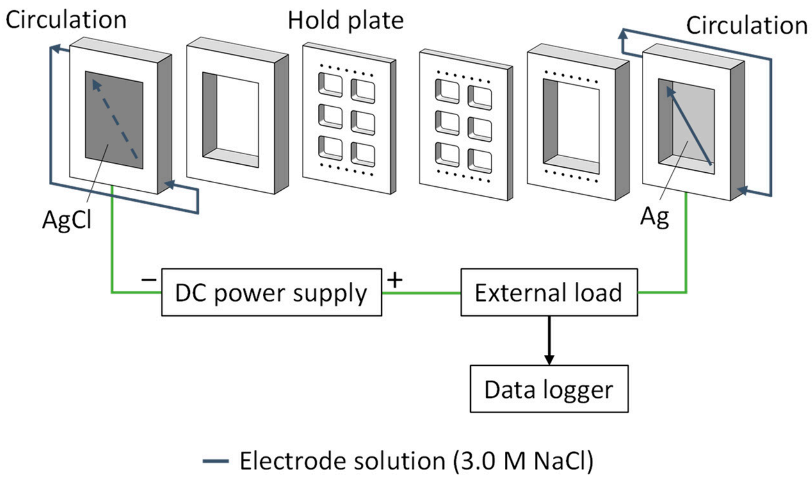

Figure S10. Set up of electrode resistance measurement.

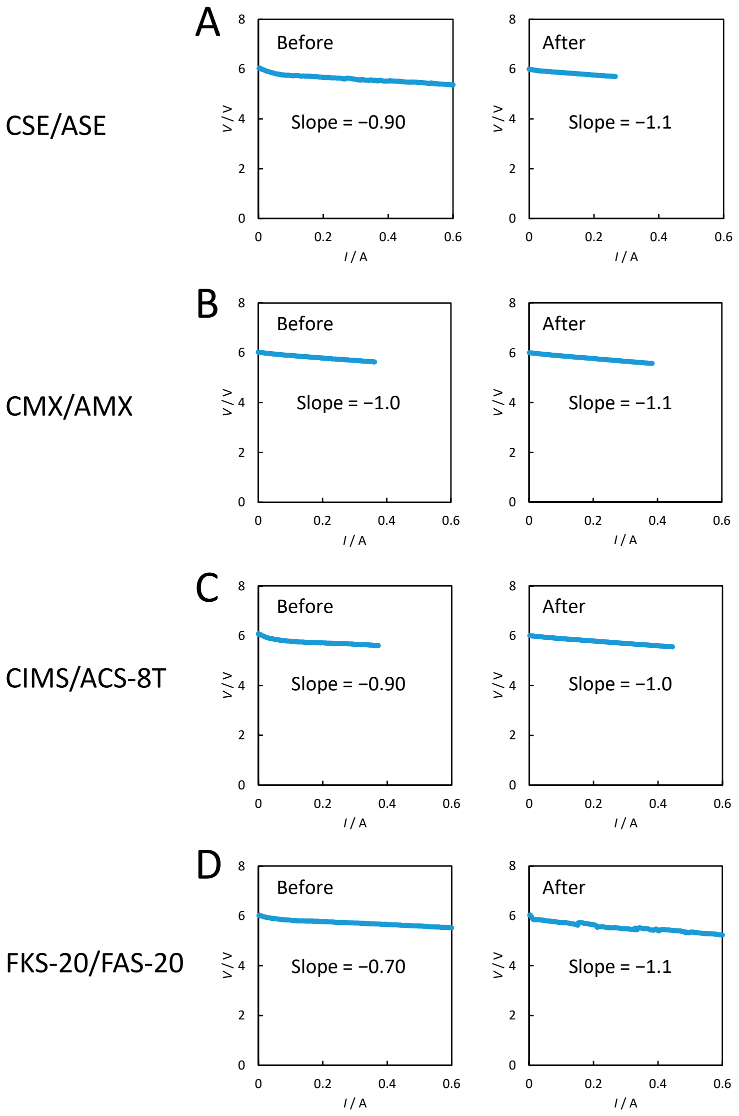

Figure S11. *V-I* curves of the stack without IEMs before and after a series of power generation test using model SW and model RW with (A) CSE/ASE, (B) CMX/AMX (C) CIMS/ACS-8T, and (D) FKS-20/FAS-20.

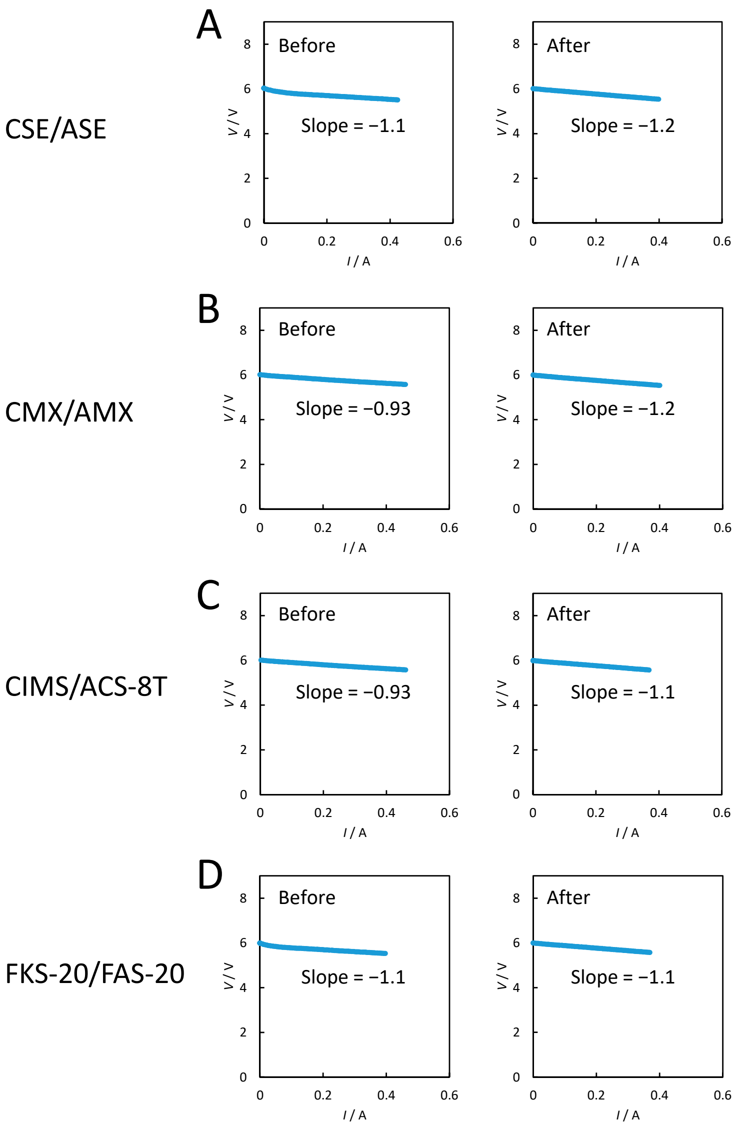

Figure S12. *V-I* curves of the stack without IEMs before and after a series of power generation test using model RO brine and model RW with (A) CSE/ASE, (B) CMX/AMX (C) CIMS/ACS-8T, and (D) FKS-20/FAS-20.

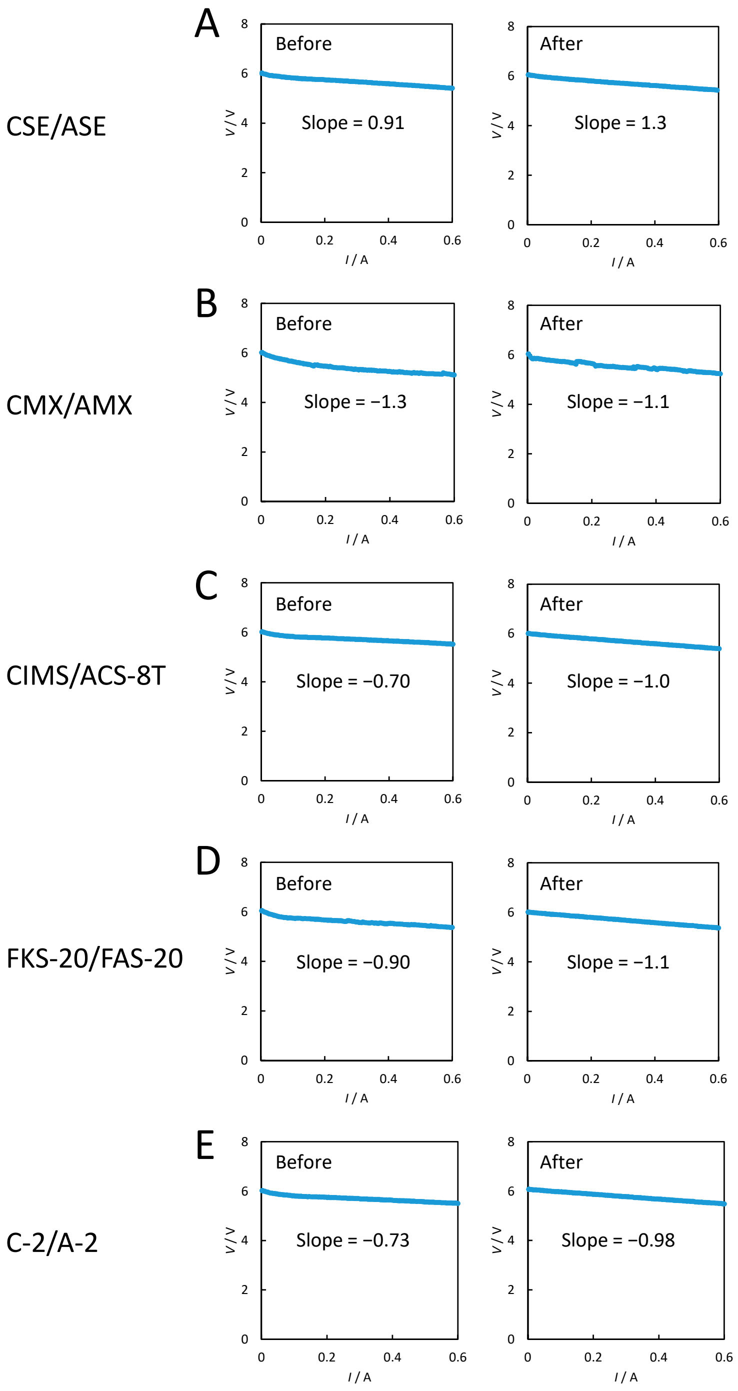

Figure S13. *V-I* curves of the stack without IEMs before and after a series of power generation test using natural SW and surface water with (A) CSE/ASE, (B) CMX/AMX (C) CIMS/ACS-8T, (D) FKS-20/FAS-20, and (E) C-2/A-2.

•Calculation of β_{sol} and β_{mem} [2]

Spacer net geometry used in this calculation is shown in Figure S14. Using the values of spacer net geometry in Figure S14, the calculation of β_{sol} was performed as follows.

$$\beta_{\mathrm{sol}} = \frac{\beta_{\mathrm{area}} + \beta_{\mathrm{vol}}}{2} \tag{S1}$$

with

$$a_{\mathrm{f}} = D_{\mathrm{W}}W + D_{\mathrm{L}}(L - D_{\mathrm{W}}) \tag{S2}$$

$$a_{\mathrm{t}} = WL\sin\theta \tag{S3}$$

$$A' = \frac{a_{\mathrm{f}}}{a_{\mathrm{t}}} \tag{S4}$$

$$\beta_{\mathrm{area}} = \frac{1}{1 - A'} \tag{S5}$$

$$v_{\mathrm{f}} = \frac{\pi\left(D_{\mathrm{W}}^2 W + D_{\mathrm{L}}^2 L\right)}{4} \tag{S6}$$

$$v_{\mathrm{t}} = a_{\mathrm{t}} t_{\mathrm{sp}} \tag{S7}$$

$$v' = v_{\mathrm{f}}/v_{\mathrm{t}} \tag{S8}$$

$$\beta_{\mathrm{vol}} = \frac{1}{1 - v'} \tag{S9}$$

where a_{f} is the filaments area per unit cell, a_{t} is the unit cell area, A' is the fiber area fraction, β_{area} is the area shadow effect, v_{f} is the filament volume of each unit cell, v_{t} is the unit cell volume, v' is the fiber volume fraction, and β_{vol} is the volume shadow effect.

The calculation of β_{mem} was performed as follows.

$$\beta_{\mathrm{mem}} = 1 + \frac{2\beta_{\mathrm{area}} D_{\mathrm{ave}}}{\alpha_i} \tag{S10}$$

with

$$D_{\mathrm{ave}} = \frac{D_{\mathrm{W}} + D_{\mathrm{L}}}{2} \tag{S11}$$

where D_{ave} is the average of horizontal and vertical filament diameters and α_{i} is the dimensional coefficient related to the membrane thickness. The value of α_{i} was set to 6.18 as described in Mehdizadeh's paper.

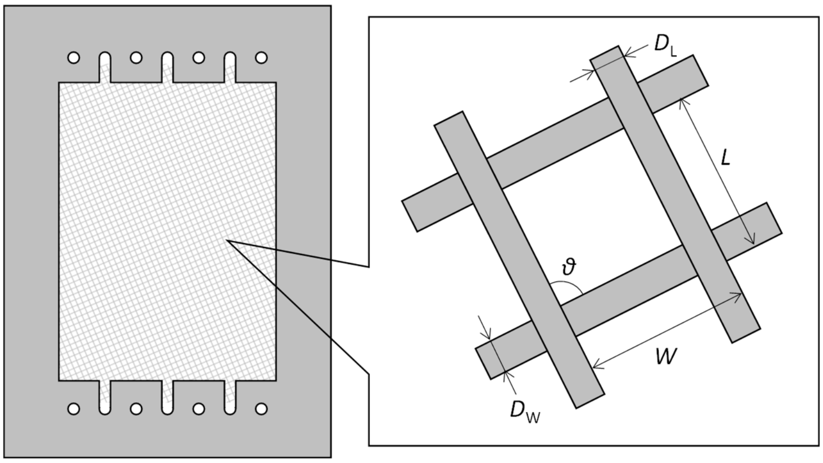

Spacer thickness (t_{sp}) = 0.199 mm
L = 0.411 mm
W = 0.409 mm
θ = 90 degree
D_L = 0.138 mm
D_W = 0.138 mm

Figure S14. Spacer net geometry in this study.

References

1. Hamer, W.J.; Wu, Y.-C. Osmotic Coefficients and Mean Activity Coefficients of Uni Univalent Electrolytes in Water at 25°C. *J. Phys. Chem. Ref. Data* **1972**, *1*, 1047–1100, doi:10.1063/1.3253108.
2. Mehdizadeh, S.; Yasukawa, M.; Abo, T.; Kakihana, Y.; Higa, M. Effect of Spacer Geometry on Membrane and Solution Compartment Resistances in Reverse Electrodialysis. *J. Membr. Sci.* **2019**, *572*, 271–280, doi:10.1016/j.memsci.2018.09.051.
